# Cooperative Anchor-Free Position Estimation for Hierarchical Wireless Sensor Networks

**DOI:** 10.3390/s100201176

**Published:** 2010-02-01

**Authors:** Chih-Yu Wen, Yu-Cheng Hsiao, Fu-Kai Chan

**Affiliations:** Department of Electrical Engineering, Graduate Institute of Communication Engineering, National Chung Hsing University, Taichung 402, Taiwan; E-Mails: shaw73324@yahoo.com.tw (Y.-C.H.); 58990006@yahoo.com.tw (F.-K.C.)

**Keywords:** anchor-free localization, cooperative estimation fusion, wireless sensor networks

## Abstract

This paper proposes a distributed algorithm for establishing connectivity and location estimation in cluster-based wireless sensor networks. The algorithm exploits the information flow while coping with distributed signal processing and the requirements of network scalability. Once the estimation procedure and communication protocol are performed, sensor clusters can be merged to establish a single global coordinate system without GPS sensors using only distance information. In order to adjust the sensor positions, the refinement schemes and cooperative fusion approaches are applied to reduce the estimation error and improve the measurement accuracy. This paper outlines the technical foundations of the localization techniques and presents the tradeoffs in algorithm design. The feasibility of the proposed schemes is shown to be effective under certain assumptions and the analysis is supported by simulation and numerical studies.

## 1. Introduction

One of the most needed and challenging components in an ad-hoc wireless network is the development of practical localization algorithms for the automatic discovery of sensor position. Due to the low power, lower cost, and simple configuration requirements of wireless sensor networks, GPS devices and the installation of a base station may be precluded. Hence, robust and distributed internal algorithms are required for sensor positioning problems.

It has been shown that cluster architecture guarantees basic performance achievement in ad-hoc networks, since this effective topology control technique conserves limited energy resources, improves energy efficiency, and further provides scalability and robustness for the network. Accordingly, we propose a distributed localization algorithm for cluster-based wireless sensor networks. This paper assumes that a number of sensors are scattered about the landscape. Initially, all of the sensor positions are unknown and must deduce their positions based on the limited information they receive. The basic strategy is to allow groups of nearby sensors to deduce their positions relative to each other in the cluster formation. These clusters are defined by their shared “local” coordinate systems. To this end, the *Cooperative Hierarchical Positioning Algorithm* (CHPA) performs location estimation in four phases: (I) Initial Local Position Estimation; (II) Position Refinement, (III) Relative Global Coordinate System, and (IV) Cooperative Estimation Fusion.

In Phase I, at the local level, sensors exploit the “particle filter” methodology [1, 2] to carry out the needed calculations. Besides the advantages of a Bayesian approach, the particles allow a robust method of location identification, which can be tailored to communicate (virtually) any amount of information between sensors. By quantifying the inherent trade-offs (cost of communication vs. improvement with increased communication), it is likely to lead to an adaptable strategy applicable in a variety of situations.

In Phase II, once a sensor has obtained the initial position estimate, due to the errors occurring in the distance estimation, the sensor needs to implement a refinement mechanism to determine its position. Assume that the *m*th sensor is located at position $x_{*}^{m}$, and the sensor’s best estimate of its current position at time *k* is $x_{k}^{m}$. The goal of the positioning refinement is to reduce the difference between the estimated locations and the real locations. This paper describes a distributed refinement scheme, which applies the Markov chain Monte Carlo (MCMC) method on each estimated sensor right after the location estimation such that estimation error and propagation error can be reduced in a distributed way.

In Phase III, a communication protocol allows nearby clusters (these which share “border sensors”) to merge into larger clusters until eventually the complete network is referred to the same coordinate system. The calculations are done in a decentralized manner since the cost of communication (in terms of power consumption) is high.

In Phase IV, based on the refined position estimates and the relative coordinate system, when the measurement does not meet the required estimation accuracy, the target sensor may broadcast a fusion message to its neighbors, group nearby two sensors into a measurement system, and trigger the cooperative sensor fusion to resolve conflicts or disagreements, and to complement the observations of the environment. This work introduces the centralized scheme, the progressive scheme, and the distributed scheme for cooperative position estimation. A centralized estimation approach is a process structure that all the neighboring sensors transmit their observations directly to the estimated sensor (the central unit) where the estimation is performed. The progressive estimation method is a processing structure in which the estimation groups sequentially update the estimation result based on each group’s local observation and partial decision from its previous groups in the sequence without sending data from all sensors to a central processing unit. For the distributed scheme, the target sensor fuses its local estimate and the estimates received from the neighborhood.

One of the unique features of our algorithm is the “sharing” of distributional data by the various sensors. This has the obvious intuitive effect of helping to make all the estimates consistent. But it may also have the effect of spreading misinformation if (for instance) a sensor malfunctions. It should be possible to include reliability measures that would effectively discard “bad” information. Hence we explore adding this feature into the basic algorithm to provide extra tolerance to sensor faults, which can be viewed as an attempt to reduce or remove error propagation.

The organization of this paper is as follows: Section 2 reviews the current literature on the sensor localization approaches. Section 3 formulates the position estimation problem and derives a hierarchical solution that relies on a cooperative self-localization protocol [3]. Then, Section 4 investigates the impact of the measurement errors and the uncertainty associated with the system model on estimation accuracy. Section 5 summarizes the performance of the proposed localization methodology. Finally, Section 6 draws conclusions and shows future research directions.

## 2. Related Work

Recent approaches to location discovery often require the availability of GPS on some reference sensors [4, 5], or assume some sensors with prior position information [6, 7]. In [8], the authors describe a centralized method using connectivity constraints and convex optimization when some number of beacon nodes are initialized with known positions. For a wireless ad-hoc network, these assumptions may not be reasonable because the information may not be available or because of the communication requirements. In [9, 10], distributed systems for GPS-free positioning in ad-hoc networks are proposed to establish a relative global coordinate system. However, the computational burden of these procedures is heavy and their communications overhead is large.

[11] presents a case study of applying particle filters to location estimation for ubiquitous computing. The performance results shows that it is practical to run particle filters on devices ranging from high-end servers to handholds. [12] provides a theoretical foundation for the problem of network localization in which some nodes know their locations and other nodes determine their locations by measuring the distances to their neighbors. Grounded graphs and graph rigidity theory are applied to construct network localization. In [13], the Cramer-Rao lower bound (CRLB) is derived for network localization. The authors argue that besides considering measurement errors, algorithmic errors should be explored in assessing localization accuracy. In [14, 15], acoustic sensor networks for a relative localization system are analyzed by reporting the accuracy achieved in the position estimation. The proposed systems are designed for those applications where objects are not restricted to a particular environment and thus one cannot depend on any external infrastructure to compute their positions. The proposed mechanisms efficiently handle multiple acoustic sources by removing false-positive errors that arise from the different propagation ranges of radio and sound.

In [9,16,17], clusters consisting of clusterheads and their cluster members are first localized in order to build local coordinate systems. Registration is then used to compute the transformations between neighboring coordinate systems such that the related global coordinate system can be established. The authors in [18] propose a cluster-based localization approach to provide efficient and scalable localization in a large and high-density network. [19] proposes to use cluster-based network topology for determining the position information of the sensor nodes. [20] describes a distributed algorithm for localizing a cluster-based sensor network in the presence of range measurement noise and avoiding flip ambiguities. However, neither algorithm provides theoretical analysis for the problem of network localization.

In [21], multidimensional scaling (MDS) is applied to perform distributed optimization for network localization. Priyantha *et al.* [22] use communication hops to estimate the network’s global layout without location information of known reference nodes. [23] uses multilateration to organize a global coordinate system from local information. Patwari *et al.* [24] use one-hop multilateration from reference nodes using both received signal strength (RSS) and time of arrival (ToA). In [25], with considering a motion model in the optimization, a maximum likelihood estimator is proposed to localize a small team of robots effectively.

In order to improve position estimates, several refinement schemes are proposed in the literature [7, 26–30] by using known sensor locations and distance measurements to neighboring sensors. [7] presents an approach called AHLoS (Ad-Hoc Localization System) that enables sensor nodes to discover their locations using a set distributed iterative algorithms. [26] presents the collaborative multilateration to enable ad-hoc deployed sensor nodes to accurately estimate their locations by using known beacon locations that are several hops away and distance measurements to neighboring nodes. To prevent error accumulation in the network, node locations are computed by setting up and solving a global non-linear optimization problem. [28] proposes a heuristic refinement approach to improve position estimates. [29] proposes an iterative quality-based localization (IQL) algorithm for location discovery. The IQL algorithm first determines an initial position estimate, after which the Weighted Least-Squares (WLS) algorithm is used iteratively to refine the position. In the WLS algorithm the Gaussian distribution is used to determine the reliability of measurements. [30] attempts to find locations for the sensors which best fit the set of all range measurements made in the network in a least-mean-squares sense. [27] demonstrates the utility of nonparametric belief propagation (NBP) for self-localization in sensor networks. However, the computational complexity and communication costs inherent in a distributed implementation of NBP are high. Comprehensive surveys of design challenges and positioning algorithms for wireless networks can be found in [31–36].

In this paper, we consider the possibility of flip ambiguity (detailed in Section 3.3.) and provide relative global localizations with measurement noise. One of the main characteristics of the proposed approach is that each sensor carries along a complete distribution of estimates of its position. It helps to solve the local minimum issues that often plague such nonlinear estimation problems by allowing the data to drive the collapse of the distribution—thus, as the data increases to the point where the position is more sure, then the distribution collapses to a point. Moreover, the distribution is inherently a measure of the accuracy of the estimation; hence if a given task requires a certain accuracy, it is possible to determine if that level of accuracy is currently available. Most importantly, the network localization is complete without absolute position information of reference nodes, which may be useful for commercial and scientific applications of wireless ad-hoc sensor networks.

Though much research has studied cooperative localization with the emphasis on algorithms [9, 16, 32, 37] very few works focus on the fundamental performance limits and GPS-free positioning in the presence of range measurement inaccuracy. This paper outlines the technical foundations of the localization techniques and presents the tradeoffs in algorithm design. A scalable distributed algorithm for sensor localization problem is proposed and an estimation-theoretic analysis of the proposed measurement mechanism is presented to assess the achievable estimation accuracy and to explore the fundamental performance of the algorithm. Specifically, a statistical model is derived to describe the localization performance considering unreliable measurements, which may provide a valuable way to show the limits of performance.

## 3. Cooperative Hierarchical Positioning Algorithm (CHPA)

This section describes a distributed algorithm that forms a relative global coordinate system efficiently. The localization operation is performed in four phases: “initial local position estimation”, “position refinement”, “relative global coordinate system”, and “cooperative estimation fusion.” The main assumptions on the network are that (a) the sensors are in fixed but unknown locations, (b) all links between sensors are bidirectional, and (c) all sensors have the same transmitting range. Observe that there is no base station or centralized control to coordinate or supervise activities among sensors.

### 3.1. Phase I: Initial Local Position Estimation

**Figure 1.**
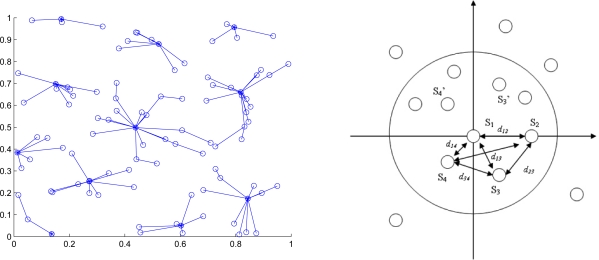
Clusters are formed in a random network of 100 sensors (left); The estimation procedures of ad-hoc wireless sensor networks in the two-dimensional space with sufficient connectivity. Sensor 1, 2 and 3 are considered as a group to form the basis for the local coordinate system. Since there are two possible locations of sensor 3, either *S*_3_ or $S_{3}^{\prime }$, two related local coordinate systems are formed due to the mirror property of the cluster (right).

When sensors of a network are first deployed, they may apply the Clustering Algorithm via Waiting Timer (CAWT) from [38] to partition the sensors into clusters using the waiting timer

(1) $${\mathrm{WT}}_{i}^{\left(k+1\right)}=\gamma \cdot {\mathrm{WT}}_{i}^{\left(k\right)}$$

where ${\mathrm{WT}}_{i}^{\left(k\right)}$ is the waiting time of sensor *i* at time step 0 < *γ* < 1 is inversely proportional to the number of neighbors. If the random waiting timer expires and none of the neighboring sensors are in a cluster, then sensor *i* declares itself a clusterhead. It then broadcasts a message notifying its neighbors that they are assigned to join the new cluster with ID *i.* After applying the CAWT, there are three different kinds of sensors: (1) the clusterheads (2) sensors with an assigned cluster ID (3) sensors without an assigned cluster ID, which will join any nearby cluster later and become 2-hop sensors. Thus, the topology of the ad-hoc network is now represented by a hierarchical collection of clusters. Figure 1 (left) shows an example of the cluster formation in a random network of 100 sensors with *R/*ℓ = 0.175, where *R* is the transmission range and ℓ is the side length of the square.

When the estimation procedure starts in a cluster-based network topology, a clusterhead called sensor 1 locates itself at the origin(0, 0) and selects the left-hand or the right-hand coordinate system as the local coordinate assignment. Then sensor 1 detects its neighbors and deploys one of the neighboring sensors, sensor 2, to the *x*-axis at (*d*_12_, 0) based on the distance information *d*_12_. A third sensor is selected to be sensor 3 which has connectivity to both sensors 1 and sensor 2. Given the known positions of sensors 1 and 2 and distance information, *d*_13_ and *d*_23_, sensor 3 can estimate its own location to the responding coordinate system. Therefore, sensors 1, 2 and 3 considered as a group form a basis for this local coordinate system. The solvability of the network localization problem is detailed in [39], which suggests that if the three known sensors are no-three-in-line, the network localization problem is solvable and the unknown position can be determined in the two-dimensional space. Accordingly, all other sensors which are within communication range of these sensors can then estimate their positions with respect to this local coordinate system. Similarly, as the cluster of known sensors grows, the location of each of the unknown sensors can be determined from three neighboring known sensors. Thus, the sensor locations can be obtained by building a local coordinate system from the clusterhead and applying multilateration to enable ad-hoc deployed sensor nodes to accurately estimate their locations by using known sensor locations and neighboring distance measurements. Figure 1 (right) shows the estimation procedures of ad-hoc wireless sensor networks in the two-dimensional space with sufficient connectivity.

Suppose that a sensor does not know its position but is able to receive information from other sensors which are assumed to have relative local position estimates. There are many ways to “solve” this sensor location problem. This section details the Bayesian particle filter method which may be preferred because it is robust to noisy measurements, it allows for flexible information transmission, and it can be robust to lost or lossy data.

Assume the *m*th sensor obtains a new measurement from (at least) three sensors and estimates its own position using the particle filter. The sensor position is given by the discrete-time state equation

(2) $$x_{k}^{m}={\Phi x}_{k-1}^{m}+{\Gamma w}_{k}$$

where $x_{k}^{m}$ is the position of the sensor and *w_k_* is an uncorrelated Gaussian diffusion term describing the uncertainty. Note that this system equation is suitable for many different systems and the only changes will be the matrixes Φ and Γ, which depend on the system model. For instance, the differences of the methodology between a moving sensor and a fixed sensor are the choices of Φ and Γ, and the rest of the methodology is the same. Hence, the same basic procedure can be used in other tasks such as target tracking. Here we assume the sensors do not move between observations, Φ is the two-dimensional identity matrix, and Γ is a zero matrix. The measurement term for the *m*th sensor is

(3) $$Z_{k}^{m}=\sum_{\ell \in I_{m}}\left|∥x_{k}^{m}-x_{k}^{\ell }∥-d_{m\ell }\right|+v_{k}$$

where the sum is over the nearby sensors $x_{k}^{\ell }$, *I_m_* is the index set of estimated known sensors, ||·|| denotes the ℓ_1_-norm ranging measurement, *d_m_*_ℓ_ represents the measured distance between the sensors and may be approximated in application by the inverse of the signal strength or by calculated from the time delay between transmission and reception [40], and the measurement noise *v_k_* is another uncorrelated zero mean Gaussian white noise process.

**Figure 2.**
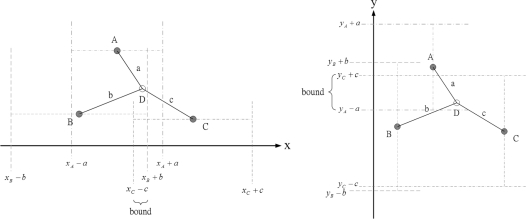
The *x* and *y* coordinate bounds of the unknown sensor can be obtained by the distance and position information, which provides a good set of initial samples for the particle filtering.

Before measurements are taken at *k* = 1, the initial state vector is obtained by applying the distance measurements as constraints on the *x* and *y* coordinates of the unknown sensor. The idea in [7], using known sensor positions and the bounding-box algorithm to extrapolate unknown sensor positions, inspires us to choose a proper prior density for generating initial samples. Figure 2 shows how the distance information can be use to obtain the *x* and *y* coordinate bounds of the unknown sensor. Therefore, the unknown sensor combines its bounds on the coordinates to form a bounding box, which provides a good set of initial samples for the particle filtering. The particle filter method is shown in Table 1.

### 3.2. Phase II: Position Refinement

Due to the error caused by the location estimation algorithm (the estimation error) and the error intrinsic to the problem (noisy distance measurements), location adjustment algorithms are needed in order to improve the estimation accuracy and limit the propagation errors. After the sensor is located near to its true position, a refinement technique is applied to elevate the estimates immediately. This subsection details the operation of a distributed model for refining the location estimates based on the initial position information from Phase I.

Because the particle filter loses diversity in the samples for static models, the Metropolis-Hastings (M-H) algorithm [41] may be used to generate new samples and provide improved estimation accuracy. The basic idea of the M-H algorithm is to simulate an ergodic Markov chain whose samples are asymptotically distributed according to the target probability distribution π(·) and use a candidate proposal distribution *q*(*x_k_*(*i*)*,* ·) to select the candidate of the current state independently with the acceptance probability

(4) $$\alpha (x_{k}(i),x_{k}^{\prime }(i))=\text{min}\left\{1,\frac{\pi (x_{k}^{\prime }(i))q(x_{k}^{\prime }(i),x_{k}(i))}{\pi (x_{k}(i))q(x_{k}(i),x_{k}^{\prime }(i))}\right\}.$$

Therefore, instead of using a centralized accumulator host to adjust sensor locations, applying the Markov chain Monte Carlo (MCMC) method on each estimated sensor right after the location estimation allows estimation error and propagation error to be reduced in a distributed way. Here we summarize the M-H algorithm with the initial value *x*_0_(*i*) in Table 2. The performance evaluation and the discussion of the proposed location adjustment algorithms are depicted in Section 5.

### 3.3. Phase III: Relative Global Localization

This section shows how the geometrical and communication requirements change when merging two coordinate systems to a single one. At some point, as the position estimation proceeds, the coverage of two coordinate systems begin to overlap, at which time they may be merged together into a single coordinate system. Eventually, all sensors have been gathered into one coordinate system and the sensor location problem is solved. If there is GPS (or other absolute measures) available, then this coordinate system can be referenced to standard measurements. If there are no GPS available, then the coordinate system is relative.

#### 3.3.1. The Information Flow

The information flow for achieving a global coordinate system in a sensor network is now described. Table 3 details the messages used to communicate between sensors. The communication protocol starts when the clusterhead sends a *Local* signal to its cluster members in order to establish a local coordinate system. When a sensor has information to share, it can broadcast an *Info* signal to its neighboring sensors. Based on the transmission and reception of the *Info* signals, sensors disseminate and obtain preliminary information such as estimates of position and distance between nearby sensors. When a sensor has location information from two coordinate systems, which means it is a shared sensor in two clusters, it sends a *Merge* signal that contains information of its estimated positions from two clusters to its neighboring sensors. After finishing the process of transmitting and receiving the *Merge* signal, the border sensors calculate adjustment information, a translation vector $\vec{d_{s}}$ and an orthonormal rotation matrix *R_merge_* (detailed in Subsection), for reorienting the coordinate system. Then it transmits an *Adjust* message that contains adjustment information to the reoriented cluster in order to convert the two coordinate systems into a single one, this merging the two clusters into one.

#### 3.3.2. Relative Global Coordinate System

**Figure 3.**
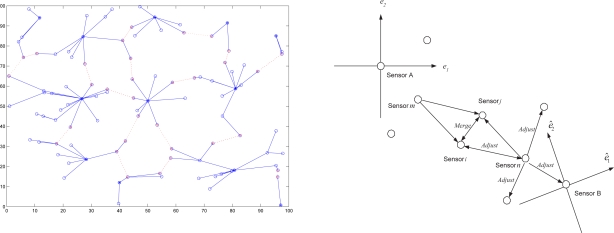
An example of the cluster formation with distributed border sensors (left); The process of merging clusters. The border sensors, sensor *i* and sensor *j*, communicate with each other by sending *Merge* messages in order to obtain adjustment information. Then sensor *i* transmits an *Adjust* signal to the sensors in the reoriented cluster (right).

Now we consider two neighboring clusters generated from clusterheads *A* and *B*. Denote the sensor which can communicate with more than one cluster as a border sensor. If there are two border sensors between cluster *A* and *B*, and if those two sensors can communicate with each other, the two clusters can be merged. This kind of network topology may be formed by applying the topology management algorithm proposed in [42]. Figure 3 (left) shows an example of the cluster formation with distributed border sensors.

The process of merging the two clusters consists of a calculation of the adjustment information and a communication protocol whereby the results of that calculation can be transmitted throughout the cluster. Figure 3 (right) illustrates the process of merging clusters by applying the communication protocol and the adjustment information in a two-dimensional space. This process of finding these adjustment quantities, $\vec{d_{s}}$ and *R_merge_*, is called coordinate system registration [43, 44]. The aim of coordinate registration is to transform a point *p* in the right-hand or left-hand coordinate system to the corresponding point *p*′ in the right-hand one applying the adjustment information: $p^{\prime }=R_{\mathrm{merge}}\cdot p+\vec{d_{s}}$.

Suppose cluster *A* and cluster *B* are adjacent and sensors *i* and *j* are two border sensors. Given that cluster *A* is in the reference right-hand coordinate system, here two cases are considered: (1) Cluster *B* is in the right-hand coordinate system; (2) Cluster B is in the left-hand coordinate system.

For case 1, based on the preliminary information, the border sensors have

(5) $${\vec{w}}_{\left(1\right)}={\left[\begin{array}{l} x_{i}\\ y_{i} \end{array}\right]}_{A}-{\left[\begin{array}{l} x_{j}\\ y_{j} \end{array}\right]}_{A}$$

(6) $${\vec{v}}_{\left(1\right)}={\left[\begin{array}{l} x_{i}\\ y_{i} \end{array}\right]}_{B}-{\left[\begin{array}{l} x_{i}\\ y_{j} \end{array}\right]}_{B}.$$

Thus, the rotation angle $\theta_{\mathrm{merge}}^{\left(1\right)}$ is

(7) $$\theta_{\mathrm{merge}}^{\left(1\right)}={\text{cos}}^{-1}\frac{{\vec{w}}_{\left(1\right)}\cdot {\vec{v}}_{\left(1\right)}}{\left|{\vec{w}}_{\left(1\right)}\right|\left|{\vec{v}}_{\left(1\right)}\right|},\;\;0\leq \theta_{\mathrm{merge}}^{\left(1\right)}\leq \pi$$

Then the orthogonal matrix $R_{\mathrm{merge}}^{\left(1\right)}$ is obtained to encapsulate the rotation operation. With the adjustment information, the transformed positions of sensors *i* and *j* yield

(8) $$p_{i}^{\prime }={\left[\begin{array}{l} x_{i}^{\prime }\\ y_{i}^{\prime } \end{array}\right]}_{A}=R_{\mathrm{merge}}^{\left(1\right)}{\left[\begin{array}{l} x_{i}\\ y_{i} \end{array}\right]}_{B}+\vec{d_{s}}$$

(9) $$p_{j}^{\prime }={\left[\begin{array}{l} x_{j}^{\prime }\\ y_{j}^{\prime } \end{array}\right]}_{A}=R_{\mathrm{merge}}^{\left(1\right)}{\left[\begin{array}{l} x_{j}\\ y_{j} \end{array}\right]}_{B}+\vec{d_{s}}$$

Accordingly, the transformation errors are given by

(10) $${\mathrm{error}}_{p_{i}^{\prime }}={\left[\begin{array}{l} x_{i}^{\prime }\\ y_{\prime }^{i} \end{array}\right]}_{A}-{\left[\begin{array}{l} x_{i}\\ y_{i} \end{array}\right]}_{A}$$

(11) $${\mathrm{error}}_{p_{j}^{\prime }}={\left[\begin{array}{c} {x^{\prime }}_{j}\\ {y^{\prime }}_{j} \end{array}\right]}_{A}-{\left[\begin{array}{l} x_{j}\\ y_{j} \end{array}\right]}_{A}$$

For case 2, the positions of sensors *i* and *j* in the coordinate system of cluster B need to be mirrored around one of their axes. That is,

(12) $$p_{i}={\left[\begin{array}{c} -x_{i}\\ y_{i} \end{array}\right]}_{B}\;\;\;\;\;\;\;\;\;\;p_{j}={\left[\begin{array}{c} -x_{j}\\ y_{j} \end{array}\right]}_{B}$$

Thus, the rotation angle $\theta_{\mathrm{merge}}^{\left(2\right)}$ is described as in (7) with

(13) $${\vec{w}}_{\left(2\right)}={\left[\begin{array}{c} x_{i}\\ y_{i} \end{array}\right]}_{A}-{\left[\begin{array}{c} x_{j}\\ y_{j} \end{array}\right]}_{A}$$

(14) $${\vec{v}}_{\left(2\right)}={\left[\begin{array}{c} -x_{i}\\ y_{i} \end{array}\right]}_{B}-{\left[\begin{array}{c} -x_{j}\\ y_{j} \end{array}\right]}_{B}$$

and the reoriented positions are

(15) $$p_{i}^{\prime \prime }={\left[\begin{array}{c} x_{i}^{\prime \prime }\\ y_{i}^{\prime \prime } \end{array}\right]}_{A}=R_{\mathrm{merge}}^{\left(2\right)}{\left[\begin{array}{c} -x_{i}\\ y_{i} \end{array}\right]}_{B}+\vec{d_{s}}$$

(16) $$p_{j}^{\prime \prime }={\left[\begin{array}{c} x_{j}^{\prime \prime }\\ y_{j}^{\prime \prime } \end{array}\right]}_{A}=R_{\mathrm{merge}}^{\left(2\right)}{\left[\begin{array}{c} -x_{j}\\ y_{j} \end{array}\right]}_{B}+\vec{d_{s}}$$

As a result, the transformation errors are

(17) $${\mathrm{error}}_{p_{i}^{\prime \prime }}={\left[\begin{array}{c} x_{i}^{\prime \prime }\\ y_{i}^{\prime \prime } \end{array}\right]}_{A}-{\left[\begin{array}{c} x_{i}\\ y_{i} \end{array}\right]}_{A}$$

(18) $${\mathrm{error}}_{p_{j}^{\prime \prime }}={\left[\begin{array}{c} x_{j}^{\prime \prime }\\ y_{j}^{\prime \prime } \end{array}\right]}_{A}-{\left[\begin{array}{c} x_{j}\\ y_{j} \end{array}\right]}_{A}$$

Given the transformation errors (10), (11), (17), and (18), the border sensors may use a criterion with local preliminary information such as neighboring connectivity to determine the relationship between the coordinate systems of two clusters. Considering the observations under the two hypotheses ${ℋ}_{1}:Z_{1}=||{\mathrm{error}}_{p_{i}^{\prime }}||+||{\mathrm{error}}_{p_{j}^{\prime }}||$ and ${ℋ}_{2}:Z_{2}=||{\mathrm{error}}_{p_{i}^{\prime \prime }}||+||{\mathrm{error}}_{p_{j}^{\prime \prime }}||$, the decision rule for the registration process becomes: ℋ_1_ : *z*_1_ < *z*_2_; ℋ_2_ : *z*_1_ > *z*_2_. Based on the decision rule, the border sensors are able to compute the transformation errors for each case and to find the desired adjustment information for reorienting the coordinate system.

**Figure 4.**
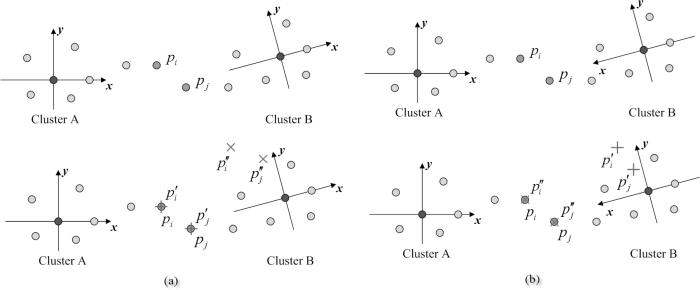
The process of coordinate system registration. (a) The right-hand coordinate hypothesis ℋ_1_: cluster *B* is in the right-hand coordinate system; (b) The left-hand coordinate hypothesis ℋ_2_: cluster *B* is in the left-hand coordinate system.

Once those calculations have been performed, sensor *i* follows the communication protocol and transmits an *Adjust* message to the sensors in the reoriented cluster in order to update their coordinates so that two local coordinate systems convert to a single one. This operation is applied repeatedly until the global coordinate system is established. Figure 4 depicts the process of coordinate registration and how the coordinate transformation is performed. To establish an absolute coordinate system, the process can proceed identically to the merging and adjusting of two clusters and follow the same communication strategy with a minimum of three GPS sensors. An example of merging two clusters is illustrated in Section 5.5.

### 3.4. Phase IV: Cooperative Estimation Fusion

**Figure 5.**
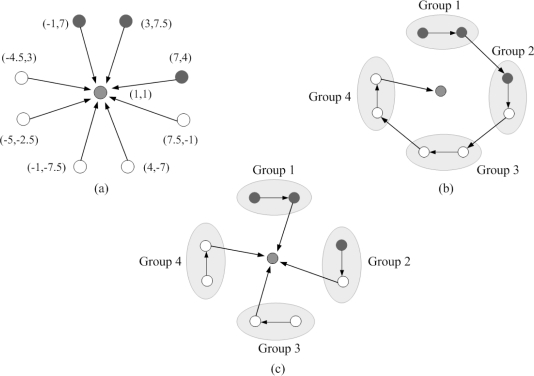
An example of group topology and the approaches for cooperative position estimation: (a) the centralized scheme, (b) the progressive scheme, and (c) the distributed scheme.

Based on the refined position estimates in Phase II and the relative coordinate system in Phase III, when the measurement does not meet the required estimation accuracy (e.g., the measurement variance is larger than the accuracy threshold), the estimated sensor, say sensor *m*, may broadcast a fusion message to its neighbors and trigger the cooperative sensor fusion to resolve conflicts or disagreements, and to complement the observations of the environment. The cooperative estimation system can be organized by sensor *m* with collecting the neighboring observations or grouping nearby two sensors into a measurement system with group IDs based on the neighboring geometric information. This subsection introduces the centralized scheme, the progressive scheme, and the distributed scheme for cooperative position estimation (Figure 5).

#### 3.4.1. The Centralized Scheme

A centralized position estimation scheme is a process structure that all the neighboring sensors transmit their observations directly to the estimated sensor (the central unit) where the estimation is performed. By means of the given measurements and (3), the approximated probability density function characterizing the cooperative estimation is obtained with the approaches in Phases I and II. The drawback is that if some sensors are faulty or the observations are corrupted, the fusion among all the neighboring sensors may deteriorate the estimation accuracy.

#### 3.4.2. The Progressive Scheme

The progressive position estimation scheme is a processing structure that the estimation groups sequentially update the estimation result based on each group’s local observation and partial decision from its previous groups in the sequence without sending data from all sensors to a central processing unit [45, 46]. Hence, only partial estimation results are transmitted through the network. In this work, the progressive scheme (Table 4) is developed based on the particle-based approaches in Phases I and II, which are used for tracking filtering and predictive distributions in the position estimation process. Each cooperative group propagates only the mean and variance of the posterior density to its next estimation group. Therefore, as shown in Figure 5, group *j* +1 may approximate the posterior density of group *j* as a Gaussian with the received mean and variance and use this Gaussian approximation [47] for the initialization of the particle filtering.

Note that this particle-based technique allows a robust method of location identification and leads to a flexible strategy for the sensing task since any amount of information can be adaptively communicated between sensors.

#### 3.4.3. The Distributed Scheme

The distributed scheme is executed in two steps: (1) Group Estimation: The position estimation is conducted within each cooperative group. Each group member sends its observation to the central unit of the cooperative group (e.g., the sensor with a higher sensor ID) where the local decision is performed. (2) Estimation Fusion: a fusion rule is applied to combine the posterior density of the estimation from each cooperative group in the estimated sensor.

Here we introduce two Bayesian fusion schemes for a distributed localization system. During the fusion process, sensor *m* fuses its local estimate and the estimates received from the neighborhood. One possible way to combine the probabilistic information obtaining from different Bayesian measurement systems is to fuse the estimates linearly [48], *i.e.*,

(19) $${\hat{\varphi }}_{m}=\sum_{\ell =0}^{N_{c}}\omega_{m\ell }\varphi_{m\ell }$$

where *N_c_* is the number of the neighboring Bayesian measurement systems in the fusion process, which is *N_c_* = *N_m_*/2 *; N_m_* is the number of neighboring sensors of sensor *m*; ω*_m_*_ℓ_ is a weight such that, 0 ≤ ω_*m*ℓ_ ≤ 1 and $\Sigma_{\ell =0}^{N_{c}}\omega_{m\ell }=1$; *ϕ*_*m*0_ is the local estimate of sensor *m*; *ϕ*_*mℓ*_ is the estimate received from the neighborhood; *ϕ̂_m_* is the fused estimate of sensor *m*.

Referring to (19), the weight reflects the significance attached to the estimate, which can be used to model the reliability of an information. As a result, the next issue is to determine ω_*m*ℓ_ for each estimate and try to weight out faulty estimates. There are many strategies to choose ω_*m*ℓ_. One scheme is to use the utility measure. Since the utility of a sensor measurement is a function of the geometric location of the sensors, here we consider the Mahalanobis measure [49]. Hence, with respect to a neighboring system estimate characterized by the mean *μ*_*m*ℓ_ and covariance Σ, the utility function for sensor *m* is defined as the geometric measure

(20) $${𝒰}_{m\ell }={\left(\mu_{m0}-\mu_{m\ell }\right)}^{T}\Sigma^{-1}(\mu_{m0}-\mu_{m\ell })$$

where *μ_m_*_0_ is the local estimated position of sensor *m* and ℓ = 1, 2, . . . , *N_c_*. That is, the utility measure is based on the Mahalanobis measure of the local estimate to a neighboring system estimate. In order to arrive at a consensus, the utility measure 𝒰*_m_*_ℓ_ can be shown to be 𝒰*_m_*_ℓ_ ≤ 1 [50].

Given the utility measure, two estimates can be allowed to be compared in a common framework and measure how much they differ *|μ_m_*_0_ *– μ_m_*_ℓ_*|*. For a larger 𝒰*_m_*_ℓ_, the neighboring system estimate may be weighted smaller, which means the weight of the estimate may be described by the inverse proportion of the utility measure. Therefore, when a neighboring system estimate succeeds the utility measure, it may cooperate with the local estimate with

(21) $$\omega_{m\ell }=\frac{\frac{1}{{𝒰}_{m\ell }}}{\sum_{k\in U_{s}}\frac{1}{{𝒰}_{mk}}}$$

where *U_s_* is the index set of the neighboring estimates that pass the utility test. Otherwise,ω*_ml_* is set to be zero. However, when the local estimate and the group estimates are non-coherent (*i.e.*, 𝒰*_ml_* *>* 1,∀ ℓ), another possible approach for sensor *m* is to choose the estimate with more confidence (less variance), or to exclude its local estimate and fuse the group estimates using the Covariance Intersection (CI) method [51]. The CI method takes a convex combination of mean and covariance estimates that are represented in information space. Since these estimates are independent, the general form is

(22) $$P_{\mathrm{cc}}^{-1}=\omega_{1}P_{a_{1}a_{1}}^{-1}+\cdots +\omega_{n}P_{a_{n}a_{n}}^{-1}$$

(23) $$P_{\mathrm{cc}}^{-1}c=\omega_{1}P_{a_{1}a_{1}}^{-1}a_{1}+\cdots +\omega_{n}P_{a_{n}a_{n}}^{-1}a_{n}$$

where $\Sigma_{i=1}^{n}\omega_{i}=1$, *n* ≥ 2, *a_i_* is the estimate of the mean from available information, *P_a_i_a_i__* is the estimate of the variance from available information, *c* is the new estimate of the mean, and *P_cc_* is the new estimate of the variance. The distributed estimation approach is summarized in Table 5.

## 4. Performance Analysis

In [22], a hardware platform (Crickets) has been developed to enable the sensor nodes to measure inter-node ranges using the time of arrival (ToA) or time difference of arrival (TDoA) between Ultrasonic and RF signals [20]. This section provides an estimation-theoretic analysis to assess the achievable ranging accuracy with the ToA information. Assume that all measurements of the arrival time stamps are independent normal random variables caused by the measurement error in the clock. This normality assumption is justified in [49] when the clock skew is small. Therefore, applying the results in [40, 52] for distance estimation with the time of arrival information, all ranging errors may be described by normal random variables. Based on the uncertainty in the distance information, the localization performance and the estimation behavior are examined. Moreover, the complexity analysis is provided to evaluate the feasibility of the proposed positioning method.

### 4.1. Analysis of Position Estimation

**Figure 6.**
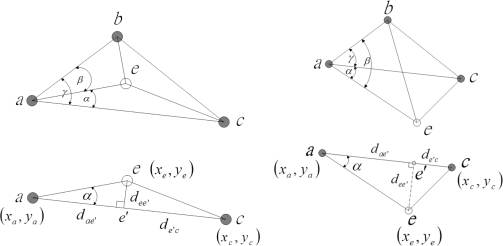
Two examples showing the way to compute the unknown sensor position (*x_e_, y_e_*) by triangulation using known distance and position information.

In order to investigate the estimation performance of the position measurement, Figure 6 depicts the measurement using known positions and distance information to obtain the unknown sensor position. Notice that sensors *a, b*, and *c* are three known sensors with estimated positions (*x_a_, y_a_*)*,* (*x_b_, y_b_*), and (*x_c_, y_c_*), respectively. Given the distance measurements, the unknown sensor position (*x_e_, y_e_*) can be computed by triangulation, which is

(24) $$x_{e}=\frac{d_{{\mathrm{ce}}^{\prime }}x_{a}+d_{{\mathrm{ae}}^{\prime }}x_{c}}{d_{{\mathrm{ae}}^{\prime }}+d_{{\mathrm{ce}}^{\prime }}}+d_{\mathrm{ae}}\left(\frac{y_{c}-y_{a}}{x_{c}-x_{a}}\right)\frac{\text{sin}\alpha }{\sqrt{1+{\left(\frac{y_{c}-y_{a}}{x_{c}-x_{a}}\right)}^{2}}}$$

(25) $$y_{e}=\frac{d_{{\mathrm{ce}}^{\prime }}y_{a}+d_{{\mathrm{ae}}^{\prime }}y_{c}}{d_{{\mathrm{ae}}^{\prime }}+d_{{\mathrm{ce}}^{\prime }}}\pm d_{\mathrm{ae}}\frac{\text{sin}\;\alpha }{\sqrt{1+{\left(\frac{y_{c}-y_{a}}{x_{c}-x_{a}}\right)}^{2}}}$$

where *d*_*ae′*_ = *d*_*ae*_ cos *α*, *d*_*ce*′_= *d_ac_* – *d*_*ae*′_,

(26) $$\alpha =\text{arccos}\left(\frac{d_{\mathrm{ae}}^{2}+d_{\mathrm{ac}}^{2}-d_{\mathrm{ce}}^{2}}{{2d}_{\mathrm{ae}}d_{\mathrm{ac}}}\right)$$

and

(27) $$\beta =\text{arccos}\left(\frac{d_{\mathrm{ab}}^{2}+d_{\mathrm{ae}}^{2}-d_{\mathrm{be}}^{2}}{{2d}_{\mathrm{ae}}d_{\mathrm{ab}}}\right)$$

Due to the errors in distance measurement, the position and distance estimates may be described by normal random variables. Let *D*_ij_ denote the normal random variable for the distance estimate between a pair of sensors *i* and *j* with $D_{\mathrm{ij}}∼N(\mu_{D_{\mathrm{ij}}},\sigma_{D_{\mathrm{ij}}}^{2})$. Let *X_i_* and *Y_i_* be the normal random variables for the known estimated *x* and *y* coordinates of sensor *i*, respectively, with $X_{i}∼N(\mu_{X_{i}},\sigma_{X_{i}}^{2})$ and $Y_{i}∼N(\mu_{Y_{i},}\sigma_{Y_{i}}^{2})$. Now we rewrite (24) as

(28) $$X_{e}=W_{1}+W_{2}$$

where the random variables

(29) $$W_{1}=\frac{D_{{\mathrm{ce}}^{\prime }}X_{a}+D_{{\mathrm{ae}}^{\prime }}X_{c}}{D_{{\mathrm{ae}}^{\prime }}+D_{{\mathrm{ce}}^{\prime }}}$$

(30) $$W_{2}=D_{\mathrm{ae}}\cdot W_{3}\cdot \frac{W_{4}}{W_{5}}$$

(31) $$W_{3}=\frac{Y_{c}-Y_{a}}{X_{c}-X_{a}}$$

(32) $$W_{4}=\text{sin}\;\alpha \;\;=\text{sin}\;\left(\text{arccos}\left(\frac{D_{\mathrm{ae}}^{2}+D_{\mathrm{ac}}^{2}-D_{\mathrm{ce}}^{2}}{2D_{\mathrm{ae}}D_{\mathrm{ac}}}\right)\right)=\sqrt{1-{\left(\frac{D_{\mathrm{ae}}^{2}+D_{\mathrm{ac}}^{2}-D_{\mathrm{ce}}^{2}}{2D_{\mathrm{ae}}D_{\mathrm{ac}}}\right)}^{2}}$$

(33) $$W_{5}=\sqrt{1+{\left(\frac{Y_{c}-Y_{a}}{X_{c}-X_{a}}\right)}^{2}}=\sqrt{1+W_{3}^{2}}$$

(34) $$W_{6}=\frac{D_{{\mathrm{ce}}^{\prime }}Y_{a}+D_{{\mathrm{ae}}^{\prime }}Y_{c}}{D_{{\mathrm{ae}}^{\prime }}+D_{{\mathrm{ce}}^{\prime }}}$$

For the random variable *W*_1_, let us first consider the distribution function of *D_ae_*_′_. Based on Figure 6, the random variable *D_ae_*_′_ is

(35) $$D_{{\mathrm{ae}}^{\prime }}=D_{\mathrm{ae}}\text{cos}\;\alpha =\frac{V}{2D_{\mathrm{ae}}}$$

where $V=D_{\mathrm{ae}}^{2}+D_{\mathrm{ac}}^{2}-D_{\mathrm{ce}}^{2}$. Thus, the random variable *V* is the weighted sum of quadratic forms of independent normal random variables (*i.e.*, a linear combination of noncentral chi-squared random variables [53,54]), which is

(36) $$V=\sum_{k=1}^{3}a_{k}U_{k}$$

where *a_k_* represents the weight and *U_k_* is an independent non-central chi-squared random variable with *p_k_* degrees of freedom and a non-centrality parameter *δ_k_*. Hence, the characteristic function of *U_k_* is

(37) $$\varphi_{U_{k}}(t)={\left(1-2it\right)}^{-\mathrm{pk}/2}\text{exp}\left(\frac{it\delta_{k}}{1-2it}\right)$$

where **i** = (−1)^1/2^, and the characteristic function of *V* is

(38) $$\varphi_{V}(t)=\prod_{k=1}^{3}\varphi_{U_{k}}(a_{k}t)$$

with the mean $\mu_{V}=\Sigma_{k=1}^{3}a_{k}(p_{k}+\delta_{k})$ and the variance $\sigma_{V}^{2}=2\Sigma_{k=1}^{3}a_{k}^{2}(p_{k}+{2\delta }_{k})$. In our case, *p_k_* = 1 ∀*k, a*_1_ = *a*_2_ = 1*, a*_3_ = −1, $\delta_{1}=\mu_{D_{\mathrm{ae}}}^{2}$, $\delta_{2}=\mu_{D_{\mathrm{ac}}}^{2}$, and $\delta_{3}=\mu_{D_{\mathrm{ce}}}^{2}$.

Referring to [55, 56], the normal approximation may be applied to obtain the distribution function, which yields

(39) $$D_{{\mathrm{ae}}^{\prime }}∼N(\mu_{D_{{\mathrm{ae}}^{\prime }}},\sigma_{D_{{\mathrm{ae}}^{\prime }}}^{2})$$

with

(40) $$\mu_{D_{{\mathrm{ae}}^{\prime }}}=\frac{\mu_{V}}{{2\mu }_{D_{\mathrm{ac}}}}$$

(41) $$\sigma_{D_{{\mathrm{ae}}^{\prime }}}^{2}=\frac{{4\sigma }_{D_{\mathrm{ac}}}^{2}\mu_{{D^{\prime }}_{\mathrm{ae}}}^{2}+\sigma_{V}^{2}}{4\mu_{D_{\mathrm{ac}}}^{2}}$$

Using the analytical techniques for normal approximation, the distribution function of *W*_1_ can be approximated by

(42) $$W_{1}∼N(\mu_{W_{1}},\sigma_{W_{1}}^{2})$$

with

(43) $$\mu_{W_{1}}=\frac{\mu_{D_{{\mathrm{ce}}^{\prime }}}\mu_{X_{a}}+\mu_{D_{{\mathrm{ae}}^{\prime }}}\mu_{X_{c}}}{\mu_{D_{{\mathrm{ae}}^{\prime }}}+\mu D_{{\mathrm{ce}}^{\prime }}}$$

(44) $$\sigma_{W_{1}}^{2}=\frac{(\sigma_{{D^{\prime }}_{\mathrm{ae}}}^{2}+\sigma_{{D^{\prime }}_{\mathrm{ce}}}^{2})\mu_{W_{1}}^{2}+\mu_{{D^{\prime }}_{\mathrm{ce}}}^{2}\sigma_{X_{a}}^{2}+\mu_{X_{a}}^{2}\sigma_{{D^{\prime }}_{\mathrm{ce}}}^{2}+\mu_{{D^{\prime }}_{\mathrm{ae}}}^{2}\sigma_{X_{c}}^{2}+\mu_{X_{c}}^{2}\sigma_{{D^{\prime }}_{\mathrm{ae}}}^{2}}{{\left(\mu_{D_{{\mathrm{ae}}^{\prime }}}+\mu_{D_{{\mathrm{ce}}^{\prime }}}\right)}^{2}}$$

In order to derive the probability density function of *W*_2_, let us first consider the random variables *W*_3_*, W*_4_, and *W*_5_, respectively.

For the random variable *W*_3_ (31), let *W*_3_ = *M*_1_/*M*_2_. Therefore, the variables *M*_1_ = *Y_c_ – Y_a_* and *M*_2_ = *X_c_* – *X_a_* follow a bivariate normal distribution $M_{1}∼N(\mu_{Y_{c}}-\mu_{Y_{a}},\sigma_{Y_{c}}^{2}+\sigma_{Y_{a}}^{2})$ and $M_{2}∼N(\mu_{X_{c}}-\mu_{X_{a}},\sigma_{X_{c}}^{2}+\sigma_{X_{a}}^{2})$. [55] and [57] show that as the ratios *μ*_*M*_1__/*σ*_*M*_2__ and *μ*_*M*_2__/*σ*_*M*_2__ increase and the probability that *M*_2_ is negative tends to zero, then the probability density function of *W*_3_ is given by

(45) $$W_{3}∼N(\mu_{W_{3}},\sigma_{W_{3}}^{2})$$

with

(46) $$\mu_{W_{3}}=\frac{\mu_{M_{1}}}{\mu_{M_{2}}}$$

(47) $$\sigma_{W_{3}}^{2}=\frac{\mu_{M_{1}}^{2}\sigma_{M_{2}}^{2}}{\mu_{M_{2}}^{4}}+\frac{\sigma_{M_{1}}^{2}}{\mu_{M_{2}}^{2}}$$

For the random variable *W*_4_ (32), it can be further expressed by $W_{4}=\sqrt{1-Q^{2}}$, where the random variable *Q* = *V/*(2*D_ae_D_ac_*) and the random variable *V* is defined as in (36). Hence, the density function of *Q* is given by

(48) $$Q∼N(\mu_{Q},\sigma_{Q}^{2})$$

with

(49) $$\mu_{Q}=\frac{\mu v}{{2\mu }_{D_{\mathrm{ae}}}\mu_{D_{\mathrm{ac}}}}$$

(50) $$\sigma_{Q}^{2}=\frac{{4\mu }_{Q}^{2}(\mu_{D_{\mathrm{ae}}}^{2}\sigma_{D_{\mathrm{ac}}}^{2}+\mu_{D_{\mathrm{ac}}}^{2}\sigma_{D_{\mathrm{ae}}}^{2})+\sigma_{V}^{2}}{{4\mu }_{D_{\mathrm{ae}}}^{2}\mu_{D_{\mathrm{ac}}}^{2}}$$

With Taylor development of *W*_4_ at*μ_Q_*, we obtain

(51) $$W_{4}\approx \sqrt{1-\mu_{Q}^{2}}-\frac{\mu_{Q}}{\sqrt{1-\mu_{Q}^{2}}}(Q-\mu_{Q})$$

Therefore, the density function of *W*_4_ is approximated by $W_{4}∼N(\mu_{W_{4},}\sigma_{W_{4}}^{2})$ with $\mu_{W_{4}}=\sqrt{1-\mu_{Q}^{2}}$ and $\sigma_{W_{4}}^{2}=\mu_{Q}^{2}\sigma_{Q}^{2}/(1-\mu_{Q}^{2})$. Similarly, for *W*_5_ defined as in (33), with Taylor development at *μ*_*W*_3__ and applying the density function of *W*_3_, the density function may be approximated by $W_{5}∼N(\mu_{W_{5},}\sigma_{W_{5}}^{2})$ with $\mu_{W_{5}}=\sqrt{1+\mu_{W_{3}}^{2}}$ and $\sigma_{W_{5}}^{2}=\mu_{W_{3}}^{2}\sigma_{W_{3}}^{2}/(1+\mu_{W_{3}}^{2})$.

Accordingly, referring to (30) and applying the normal approximation techniques, the density function of the random variable *W*_2_ yields

(52) $$W_{2}∼N(\mu_{W_{2},}\sigma_{W_{2}}^{2})$$

with

(53) $$\mu_{W_{2}}=\frac{\mu_{D_{\mathrm{ae}}}\mu_{W_{3}}\mu_{W_{4}}}{\mu_{W_{5}}}$$

(54) $$\sigma_{W_{2}}^{2}=\mu_{D_{\mathrm{ae}}}^{2}\mu_{W_{3}}^{2}\left(\frac{\sigma_{W_{5}}^{2}\mu_{W_{4}}^{2}}{\mu_{W_{5}}^{4}}+\frac{\sigma_{W_{4}}^{2}}{\mu_{W_{5}}^{2}}\right)+\frac{\mu_{W_{4}}^{2}}{\mu_{W_{5}}^{2}}\left(\mu_{D_{\mathrm{ae}}}^{2}\sigma_{W_{3}}^{2}+\mu_{W_{3}}^{2}\sigma_{D_{\mathrm{ae}}}^{2}\right)$$

Thus, the density function of the estimated *x* coordinate *X_e_* is $X_{e}∼N(\mu_{X_{e},}\sigma_{X_{e}}^{2})$ with *μ_X_e__* = *μ*_*W*_1__ + *μ*_*W*_2__ and $\sigma_{X_{e}}^{2}=\sigma_{W_{1}}^{2}+\sigma_{W_{2}}^{2}$, where *μ*_*W*_1__, $\sigma_{W_{1}}^{2}$, *μ*_*W*_2__, and $\sigma_{W_{2}}^{2}$ are described as in (43), (44), (53), and (54), respectively.

For the estimated *y* coordinate *Y_e_*, it can be rewritten as

(55) $$Y_{e}=W_{6}\pm D_{\mathrm{ae}}\cdot \frac{W_{4}}{W_{5}}$$

Following the same analysis procedures for *X_e_* and applying the normal approximations, the density function yields

(56) $$Y_{e}∼N(\mu_{Y_{e},}\sigma_{Y_{e}}^{2})$$

with

(57) $$\mu_{Y_{e}}=\mu_{W_{6}}\pm \mu_{D_{\mathrm{ae}}}\frac{\mu_{W_{4}}}{\mu_{W_{5}}}$$

(58) $$\sigma_{Y_{e}}^{2}=\sigma_{W_{6}}^{2}+\mu_{D_{\mathrm{ae}}}^{2}\left(\frac{\sigma_{W_{5}}^{2}\mu_{W_{4}}^{2}}{\mu_{W_{5}}^{4}}+\frac{\sigma_{W_{4}}^{2}}{\mu_{W_{5}}^{2}}\right)+\frac{\sigma_{D_{\mathrm{ae}}}^{2}\mu_{W_{4}}^{2}}{\mu_{W_{5}}^{2}}$$

where the distribution of *W*_6_ yields the same results as described for *W*_1_ by substituting *Y_a_* and *Y_c_* for *X_a_* and *X_c_* in (43) and (44), respectively.

Accordingly, the localization performance can be assessed with the uncertainty in the distance and position information. Moreover, based on the above analysis, the effect of error propagation with imperfect position and distance information may be approximately depicted. Note that this analysis applies normal approximations to describe the probability density functions of the position estimation. The numerical results will be illustrated in Section 5. in order to compare the simulation results and validate the appropriateness of normal approximations.

### 4.2. Analysis of Measurement Performance

The relationship of mapping between the state and observations is not known precisely because of the measurement errors and the uncertainty associated with the system model. In order to evaluate estimation behavior, the distribution of the measurement term (3) for sensor *m* is derived to extract information about estimation accuracy.

To proceed with the analysis, notations and assumptions are introduced to capture the sensible measurement performance. We rewrite the measurement term (3) as

(59) $$Z^{m}=\sum_{\ell \in I_{m}}\left|||P^{m}-P^{\ell }||-D_{m\ell }\right|+V^{m}$$

(60) $$=\sum_{\ell \in I_{m}}\left|{\hat{D}}_{m\ell }-D_{m\ell }\right|+V^{m}$$

where *Z^m^* is the random measurement term, *I_m_* is the index set of estimated known sensors, *P^m^* is the estimated position of sensor *m*, *P*^ℓ^ is the estimated position of sensor ℓ, *D̂_mℓ_* denotes the ℓ_1_-norm ranging measurement obtained from estimated positions,*D*_*m*ℓ_ is the ranging measurement between sensors *m* and ℓ, and *V^m^* is the measurement noise. Suppose that *Z^m^*, *P^m^*, *P*^ℓ^, *D*_*m*ℓ_, and *V^m^* are assumed to be normal random variables which distributions are $Z^{m}∼N(\mu_{Z^{m}},\sigma_{Z^{m}}^{2})$, $P^{m}∼N(\mu_{P^{m}},\sigma_{P^{m}}^{2})$, $P^{\ell }∼N(\mu_{P^{\ell }},\sigma_{P^{\ell }}^{2})$, ${\hat{D}}_{m\ell }∼N(\mu_{{\hat{D}}_{m\ell }},\sigma_{{\hat{D}}_{m\ell }}^{2})$, $D_{m\ell }∼N(\mu_{D_{m\ell }},\sigma_{D_{m\ell }}^{2})$, and $V^{m}∼N(\mu_{V^{m}},\sigma_{V^{m}}^{2})$.

Now denote *F_m_*_ℓ_ as

(61) $$F_{m\ell }=\left|{\hat{D}}_{m\ell }-D_{m\ell }\right|=\left|G_{m\ell }\right|$$

where *G*_*m*ℓ_ = *D̂*_*m*ℓ_ – *D*_*m*ℓ_, which is a normal random variable with the distribution $G_{m\ell }∼N(\mu_{G_{m\ell }},\sigma_{G_{m\ell }}^{2})$ with mean *μ*_*G*_*m*ℓ__ = *μ*_*D̂*_*m*__ – *μ*_*D*_*m*ℓ__ and variance $\sigma_{G_{m\ell }}^{2}=\sigma_{{\hat{D}}_{m\ell }}^{2}+\sigma_{D_{m\ell }}^{2}=\sigma_{P^{m}}^{2}+\sigma_{p^{\ell }}^{2}+\sigma_{D_{m\ell }}^{2}$ Consider now the situation where *F_m_*_ℓ_ = *|G_m_*_ℓ_*|*. Therefore, the distribution of the absolute measurement *F_m_*_ℓ_ is described as the folded normal distribution [58]. The probability density function of the resulting folded normal distribution is

(62) $$h_{F_{m\ell }}(f_{m\ell })=\frac{1}{\sqrt{2\pi }\sigma_{G_{m\ell }}}\left[e^{-{\left(f_{m\ell }-\mu_{G_{m\ell }}\right)}^{2}/2\sigma_{G_{ml}}^{2}}+e^{-{\left(f_{m\ell }+\mu_{G_{m\ell }}\right)}^{2}/2\sigma_{G_{m\ell }}^{2}}\right],\;\;\;\;\;\;f_{m\ell }\geq 0\;$$

with mean

(63) $$\mu_{F_{m\ell }}=\sqrt{\frac{2}{\pi }}\sigma_{G_{m\ell }}e^{-\mu_{G_{m\ell }}/2_{G_{m\ell }}^{2}}+\mu_{G_{m\ell }}[1-2M(-\mu_{G_{m\ell }}/\sigma_{G_{m\ell }})]$$

and variance

(64) $$\sigma_{F_{m\ell }}^{2}=\mu_{G_{m\ell }}^{2}+\sigma_{G_{m\ell }}^{2}-\mu_{F_{m\ell }}^{2}$$

where

(65) $$M(a)=\frac{1}{\sqrt{2\pi }}\int_{-\infty }^{a}e^{-t^{2}/2}\mathrm{dt}$$

In order to estimate the position, at least three nearby location-aware sensors are needed. Without loss of generality, let the neighboring sensor ID be ℓ = 1, 2, and 3 and assume that the folded normal random variables are independent, which is a reasonable condition in our case. Thus, the distribution of the sum of the folded normals $S_{m}=\sum_{\ell =1}^{3}F_{m\ell }$ can be derived by convolution, which is

(66) $$h_{S_{m}}(s_{m})=\frac{2}{\pi }\sqrt{\frac{1}{(\sigma_{F_{m1}}^{2}+\sigma_{F_{m2}}^{2})\sigma_{F_{m3}}^{2}}}\int_{0}^{s_{m}}e^{A(x)}[\text{erf(}\sigma_{F_{m1}}B(x))+\text{erf}(\sigma_{F_{m2}}B(x))]\;dx,\;s_{m}\geq 0$$

with $A(x)=-x^{2}/2\left(\sigma_{F_{m1}}^{2}+\sigma_{F_{m2}}^{2}\right)+{\left(s_{m}-x\right)}^{2}/2\sigma_{F_{m3}}^{2}$ and $B(x)=x/\sqrt{2\sigma_{F_{m1}}^{2}\sigma_{F_{m2}}^{2}\left(\sigma_{F_{m1}}^{2}+\sigma_{F_{m2}}^{2}\right)}$, where erf(·) is the error function and $\sigma_{F_{m\ell }}^{2}$ is described as in (64), which is related to the variances of position and distance estimates.

Suppose that the measurement noise is negligible. Thus the distribution of the random measurement term *Z^m^* may be described by (66), which highly depends on the deviations of estimated anchor positions and estimated distances. Observe that the measurement term *Z^m^* is actually a way to depict estimation accuracy of the system since the objective is to minimize the estimation error. Based on the above settings, (66) may also represent an ideal measurement performance for realistic estimation experiments. However, with the deviations of distance estimates, in order to suppress the measurement error we may obtain inaccurate localization results though using the anchor sensors with accurate position information. In other words, when applying the particle filtering methodology, the measurement term *Z^m^* of sensor *m* may weight out the best possible particles due to the incorrect distance information. The measurement performance will be illustrated and further discussed in Section 5.

### 4.3. Complexity Analysis

When developing the local position estimation (Phase I), one round of local flooding is initiated in order to gather distance information. Then, the distance and position information from the nearby three nodes are applied to estimate its own position. Thus, the time complexity is 𝒪(1) round.

Suppose that the total power requirements include the power required to transmit messages *E_T_*, the power required to receive *E_R_*, and the power required to process *E_P_*. Therefore, the total energy consumption for initial local positioning in the network is $E_{L}=N_{T}^{\left(L\right)}\cdot E_{T}+N_{R}^{\left(L\right)}\cdot E_{R}+N_{P}^{\left(L\right)}\cdot E_{P}$ with

(67) $$N_{T}^{\left(L\right)}=N_{S},\;\;\;\;N_{R}^{\left(L\right)}=\sum_{i=1}^{N_{S}}N_{i},\;\;\;N_{P}^{\left(L\right)}=N_{S}$$

where *N_S_* is the number of sensors in the network and *N_i_* is the number of the neighboring sensors of sensor *i*. Thus, for a sensor node, the average energy consumption yields *E_L_/N_S_*. Given the energy consumption analysis above, the communication complexity for establishing the local coordinate system in the network and for estimating the position of a sensor node are 𝒪(*N_S_*) and 𝒪(1), respectively.

Due to the error caused by the location estimation algorithm (the estimation error) and the error intrinsic to the problem (noisy distance measurements), location adjustment algorithms (Phase II) are needed in order to improve the estimation accuracy and limit the propagation errors. Once the position refinement is executed, one round of 1-hop flooding is performed for broadcasting the estimated position information. Hence, the time complexity and communication complexity are the same as those in Phase I.

For merging the coordinate systems of two clusters (Phase III), two border sensors (sensor *p* and sensor *q*) execute one round of local flooding for exchanging the *Merge* message and determining the merging cluster. Then, the border sensor, say sensor *q*, calculates the adjustment quantities and 4 rounds of local flooding are performed for the the process of coordinate registration and transformation in the 2-hop cluster topology. Therefore, the time complexity is 𝒪(5) rounds. Based on the operation of building the relative global localization, the energy consumption yields $E_{G}=N_{T}^{\left(G\right)}\cdot E_{T}+N_{R}^{\left(G\right)}\cdot E_{R}+N_{P}^{\left(G\right)}\cdot E_{P}$ with

(68) $$N_{T}^{\left(G\right)}=(N_{ch}-1)\cdot (5+|H_{i}^{\left(2\right)}|)$$

(69) $$N_{R}^{\left(G\right)}=(N_{ch}-1)\cdot (N_{P}+2\cdot N_{q}+N_{i}+\;\sum_{k\in H_{i}^{\left(2\right)}}N_{k}+N_{r})$$

(70) $$N_{P}^{\left(G\right)}=N_{ch}-1$$

where $H_{i}^{\left(2\right)}$ denotes the index set of 1-hop cluster members of cluster *i* with neighboring 2-hop cluster members, sensor *r* is the parent node of sensor*q*, *N_i_* is the number of the neighboring sensors of sensor *i*, and *N_ch_* is the number of clusters in the network. Accordingly, the communication complexity due to performing coordinate registration and transformation is 𝒪((*N_ch_* *–* 1)*N_avg_*), where *N_avg_* is the average number of neighbors of a sensor in the network.

Based on the operations described in Section 3.4, the complexity analysis of the proposed cooperative estimation approaches (Phase IV) are described, respectively. For the centralized scheme, all the neighboring sensors transmit their observations directly to the estimated sensor. Therefore, the time complexity is 𝒪(1) round. The energy consumption yields $E_{CC}=N_{T}^{\left(C\right)}\cdot E_{T}+N_{R}^{\left(C\right)}\cdot E_{R}+N_{P}^{\left(C\right)}\cdot E_{P}$ with

(71) $$N_{T}^{\left(C\right)}=N_{i},\;\;N_{R}^{\left(C\right)}=\sum_{j\in S_{b}^{\left(i\right)}}N_{j},\;\;N_{P}^{\left(C\right)}=1$$

where $S_{b}^{\left(i\right)}$ is the index set of neighboring sensors of sensor *i*. Thus, the communication complexity due to transmitting the observations is 𝒪(*N_avg_*).

For the progressive scheme, the estimation groups update the estimation result sequentially based on each group’s local observation and partial decision from it’s previous groups in the sequence. Hence, the time complexity is 𝒪(2*N_EG_*) rounds for estimation groups consisting of two sensors, where *N_EG_* is the number of estimation groups. The energy consumption is given by $E_{CP}=N_{T}^{\left(P\right)}\cdot E_{T}+N_{R}^{\left(P\right)}\cdot E_{R}+N_{P}^{\left(P\right)}\cdot E_{P}$ with

(72) $$N_{T}^{\left(P\right)}=2\cdot N_{EG},\;\;\;N_{R}^{\left(P\right)}=\sum_{j\in G_{b}^{\left(i\right)}}N_{j},\;\;\;N_{P}^{\left(P\right)}=N_{EG}$$

where $G_{b}^{\left(i\right)}$ is the index set of sensors in the estimation groups of sensor *i*. Thus, the communication complexity due to transmitting the observations and partial decisions is 𝒪(2*N_EG_*).

For the distributed scheme, the target sensor fuses its local estimate and the estimates received from the neighborhood. Hence, the time complexity is 𝒪(2) rounds, which are for group estimation and estimation fusion. The energy consumption is given by $E_{CD}=N_{T}^{\left(D\right)}\cdot E_{T}+N_{R}^{\left(D\right)}\cdot E_{R}+N_{P}^{\left(D\right)}\cdot E_{P}$ with

(73) $$N_{T}^{\left(D\right)}=2\cdot N_{EG},\;\;\;N_{R}^{\left(D\right)}=\sum_{j\in G_{b}^{\left(i\right)}}N_{j},\;\;\;N_{P}^{\left(D\right)}=N_{EG}+1$$

Similar to the progressive scheme, the communication complexity due to transmitting the observations and group decisions is 𝒪(2*N_EG_*).

## 5. Experiments and Discussion

In order to assess the performance of the proposed methodology, the feasibility of the proposed schemes is examined via simulation and numerical results. In the following experiments, the particle filtering methodology is applied with the number of samples *N* = 500.

### 5.1. Initial Position Estimation

**Figure 7.**
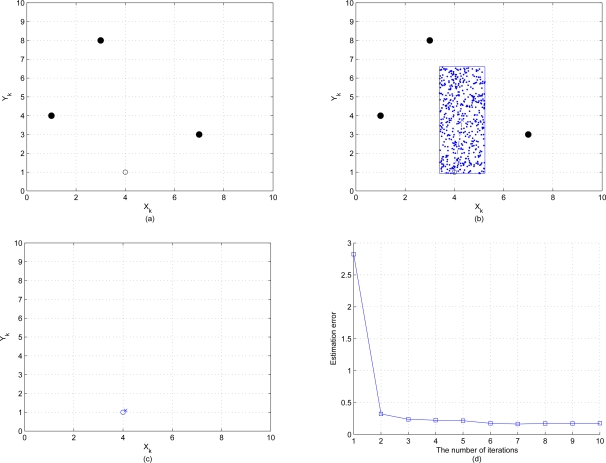
(a)–(c) are the procedures of initial position estimation by using particle filter method with bounding box algorithm; (d) shows the estimation error of the initial position estimate.

**Figure 8.**
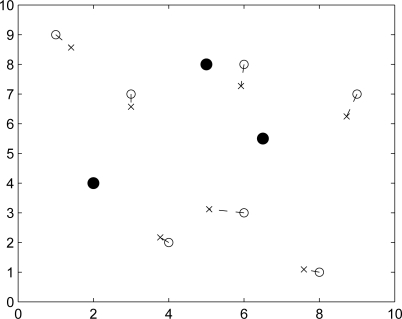
Using particle filtering for initial position estimation. “•” represents the reference node, “○” represents the unknown node, and “×” represents the initial estimated sensor location.

In this section we present the result of initial local position estimation by placing three reference nodes and one unknown node over the sensing field 10 × 10 units in size and assuming that this unknown node connects with all reference nodes. The positions of the three reference nodes are (7,3), (3,8), (1,4) and the true position of this unknown node is (4,1). Figures 7 (a)–(c) show the procedures of initial position estimate by using particle filter method with the bounding box algorithm. “•” represents the reference node, “○” represents the unknown node, “·” represents the particle and “×” represents the initial estimated sensor location. The estimation error of the initial position estimate is shown in Figure 7 (d), which depicts that the estimation error converges to 0.5 quickly after a few iterations. Figure 8 shows the resulting initial position estimation for a network of 25 nodes with 3 reference nodes, which suggests that the particle filter method with bounding box algorithm provides an acceptable performance of initial position estimation.

### 5.2. Performance of Theoretical Approximation

This set of experiments compares the initial position estimation of an unknown sensor using particle filtering and the theoretical approximation derived in Section 4.2. We consider the uncertainty of position estimation and distance measurement and then use the estimated distributions from other known sensors to get the distribution of the position estimate of the unknown sensor. Let *σ_D_* and *σ_P_* denote the distance deviation and position deviation, respectively. Suppose that the true position of the unknown sensor *m* is (17,30) and the position distributions of three known sensors are $N(\mu_{P^{1}},\sigma_{P^{1}}^{2})$, $N(\mu_{P^{2}},\sigma_{P^{2}}^{2})$, and $N(\mu_{P^{3}},\sigma_{P^{3}}^{2})$ with *μ*_*P*^1^_ = (0, 0), *μ*_*P*^2^_ = (23, 70), *μ*_*P*^3^_ = (50, 0), *σ*_*D*_*m*1__ = *σ*_*D*_*m*2__ = *σ*_*D*_*m*3__ = *σ*_*D*_, and *σ*_*P*^1^_ = *σ*_*P*^2^_ = *σ*_*P*^3^_ = *σ_P_*. In the simulation, we sample the distributions of the position estimates obtained from three known sensors and the distributions of the distance estimates 200 times in order to get the possible values for the particle filtering. Then we fuse these 200 distributions to get the possible position distribution of the unknown sensor using the Covariance Intersection (CI) method described as in Equations (22) and (23). In this set of experiments, we choose to weight each typical run equally.

**Figure 9.**
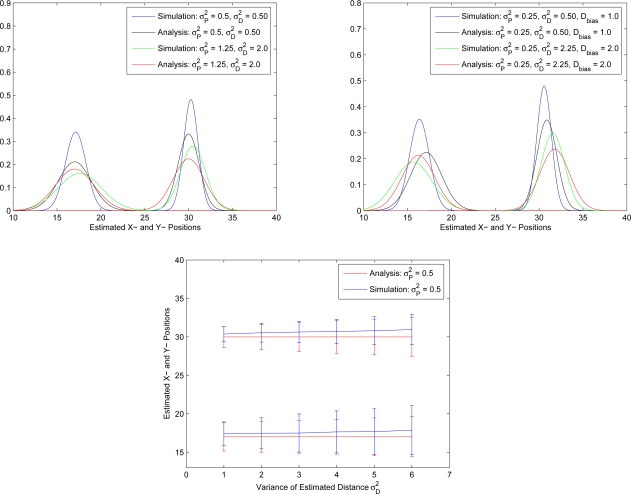
The distributions of simulation results and the theoretical approximation of initial position estimates with unbiased distance and position information (top left) and biased measurement information (top right); the estimated sensor position versus the variance of the distance measurement for simulation results and theoretical approximation (bottom).

Given the deviations at distance and position information, Figure 9 shows the distributions of simulation results and the theoretical approximation of initial position estimates with unbiased distance and position information (top left) and biased distance measurements (top right), respectively. Figure 9 (bottom) shows the standard deviation of the mean value of position estimates. The plot varies the variance of the distance measurement. Observe that Figures 9 demonstrates that the approximated probability density function can well describe the measurement performance, which implies that the approximation may be a sensible way to assess the estimation process of an unknown sensor under the circumstances with ranging and positioning errors of known sensors.

### 5.3. Measurement Performance

**Figure 10.**
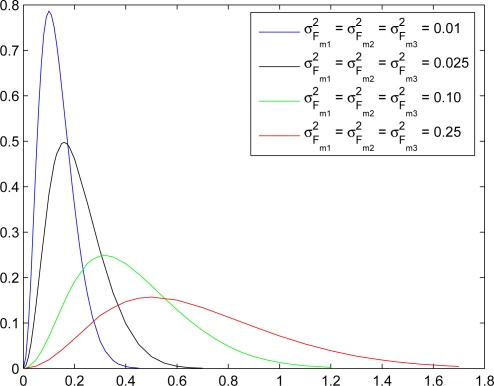
The distributions of random measurements with position and distance deviations.

Due to the errors occurring in the distance estimation, the following special case is studied to explore the impact of the ranging error on the performance of random measurement term. Assume that the mean value of the distance estimation is the true distance and the neighboring reference sensors have unbiased position information, which means the distance estimation is unbiased and the ℓ_1_-norm distance is *μ*_*D̂*_*mℓ*__ = *μ*_*D*_*mℓ*__ = *d_mℓ_* (*i.e.*, *μ*_*G*_*mℓ*__ = 0). Figure 10 shows the distributions of the random measurements with position and distance deviations. Note that as substituting the true position of the estimated sensor into the measurement term (66) with unbiased distance and position estimates having small deviations, the peak value of the distribution locates near zero, which may represent an ideal measurement case since the estimated sensor owns accurate distance and position information. On the other hand, with distance and position estimates having large deviations, the true position of the estimated sensor makes the peak value of the distribution shift away from zero. Therefore, in order to suppress the estimation error and shift the peak value of the distribution back to zero, incorrect estimates may be obtained. That is, as the iterative process of the particle filter proceeds, the most possible estimates may be weighted smaller or even be weighted out due to the low value of the likelihood function at the particle and the uncertainty of distance and position information.

### 5.4. Performance of Position Refinement

In our simulations, two proposal densities are chosen for refining the position estimates with the Metropolis-Hastings Algorithm. Proposal density I is a normal distribution centered around the current state estimate, $𝒩({\bar{x}}_{k},\sigma_{ɛ1}^{2})$. Proposal density II is the sum of all the current samples and noise. The distribution of the noise is $𝒩(0,\sigma_{ɛ2}^{2})$. Assume that $\sigma_{ɛ1}^{2}=\sigma_{ɛ2}^{2}$. This subsection reports the performance of the sensor location estimate and adjustment by applying the particle filtering and a few Monte Carlo Markov Chain (MCMC) steps on each particle. Moreover, the importance of resampling after using the MCMC steps and the impact of error propagation on estimation accuracy are explored via simulation.

#### 5.4.1. Proposal Density I

**Figure 11.**
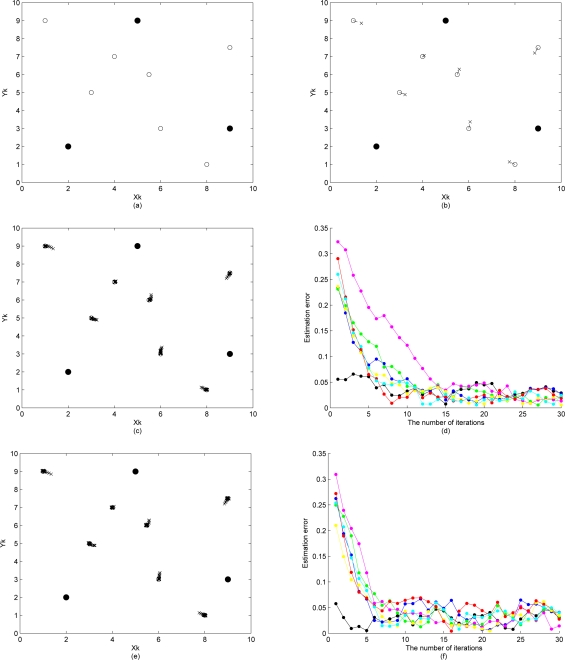
$\sigma_{ɛ1}^{2}=\sigma_{ɛ2}^{2}=0.5$ and $\sigma_{d}^{2}=0$. (a)(b) A typical simulation run of the sensor location algorithm locates all *n* = 10 sensors using distance information; (c)(d) The sensor location adjustment and estimation error of the typical simulation run by applying the Metropolis-Hastings algorithm with proposal density I; (e)(f) The sensor location adjustment and estimation error of the typical simulation run by applying the Metropolis-Hastings algorithm with proposal density II.

By adopting this density, the proposed particles are generated from a random walk $x_{k}^{\prime }(i)=x_{k}(i)+\sigma_{ɛ1}$ (random walk Metropolis-Hastings). From the work in [59], it recommends that if the proposal density is normal, then the acceptance rate should be around 0.45 for the random walk chain. Here, we adjust the parameters to achieve an acceptance rate of 0.4 to 0.5. Figures 11 (a,b) demonstrate the initial position estimation with perfect distance measurements ( $\sigma_{d}^{2}=0$). The simulation results of the sensor location estimation and the estimation error are shown in Figure 11 (c), which shows the ability of the sensor location algorithm detailed in Section 3.2. to locate all *n* = 10 sensors using only distance (e.g., received signal strength or time-of-arrival) information. In Figure 11 (d), the error propagation within the network is shown to be suppressed by applying the Metropolis-Hastings algorithm. As the number of iteration increases, a high acceptance rate will be achieved. In this case, accepted particles are taking distinct but very close values compared with the current state estimate.

#### 5.4.2. Proposal Density II

Instead of using only information from the current state estimate, the complete current distribution of samples may be used to generate the proposed particles. To evaluate the performance, the same network layout and the number of samples as that of the system with proposal density I are used. Figures 11 (e,f) illustrate the performance of the sensor location algorithm with proposal density II. Compared with Figure 11(d,f) demonstrates that using proposal density II gives more precise sensor location estimates, less estimation error, and less error propagation. In addition, a faster speed of convergence in location estimate is achieved. This is attributed to the fact that more sampling diversity is introduced and the sample impoverishment problem is alleviated.

#### 5.4.3. Resampling

**Figure 12.**
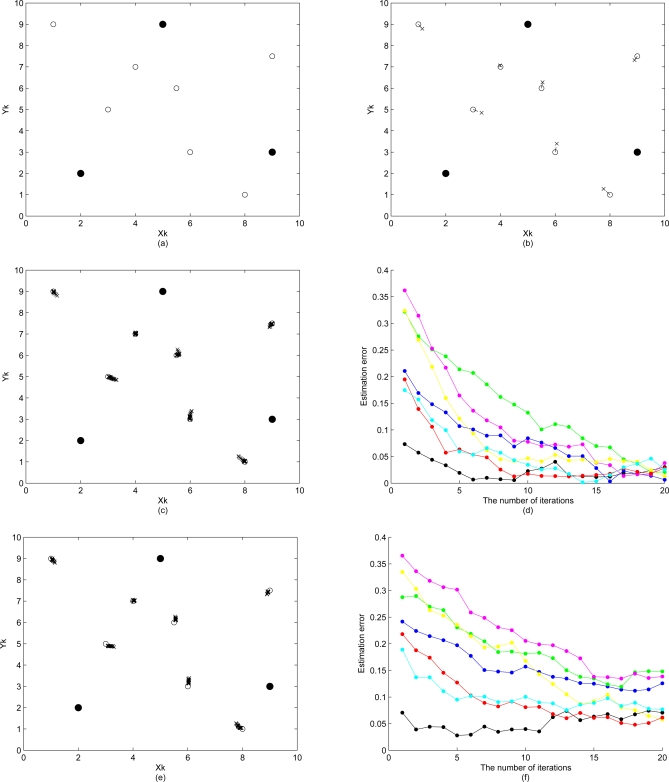
(a)(b) demonstrate the initial position estimation; (c)(d) show the sensor location adjustment and the estimation error by using distance information and proposal density I without resampling; (e)(f) show the sensor location adjustment and the estimation error by using distance information and proposal density II without resampling, where $\sigma_{d}^{2}=0$ and $\sigma_{ɛ}^{2}=0.5$.

The key point with resampling is to prevent a high concentration of probability mass at a few particles. Here we investigate the importance of resampling after carrying out the MCMC steps. For comparison, a particle filter using the same network structure and the number of particles is applied to the samples drawn from proposal density I with resampling, proposal density I without resampling, proposal density II with resampling, and proposal density II without resampling, respectively. The simulations based on these different approaches are shown in Figures 11 and 12. In these tests, the performances of the methods applying resampling (Figures 11 (d,f) are superior to those of approaches without resampling (Figures 12 (d,f)). This shows how the degeneracy problem with the MCMC steps can be reduced by using a resampling scheme.

#### 5.4.4. Error Propagation

**Figure 13.**
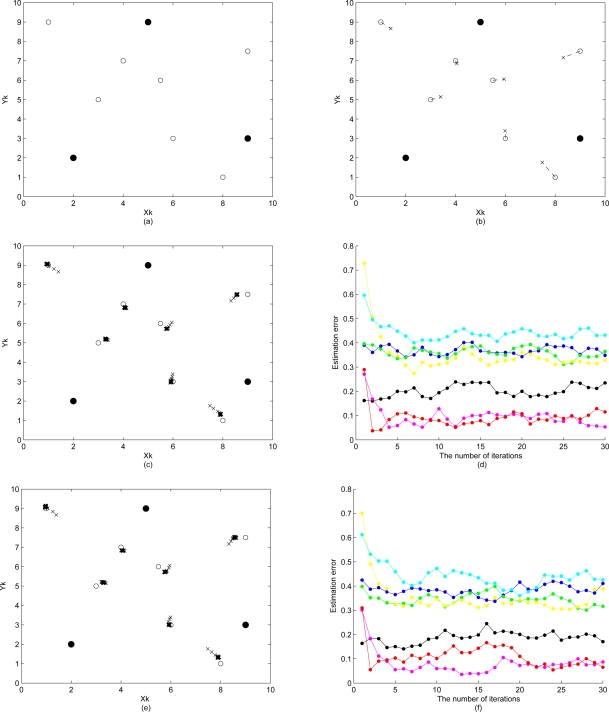
(a)(b) demonstrate the initial position estimation; (c)(d) show the sensor location adjustment and the estimation error by using distance information and proposal density I; (e)(f) show the sensor location adjustment and the estimation error by using distance information and proposal density II, where $\sigma_{d}^{2}=0.1$ and $\sigma_{ɛ}^{2}=0.5$.

Cluster-based positioning methods may introduce poor error propagation characteristics due to the lack of absolute reference points in the hierarchical network topology. Thus, the impact of error propagation on estimation accuracy is explored given the measurement errors. In the proposed approach, the amount of error propagated over the cluster is reduced based on the refinement scheme. Figures 13 (a,b) demonstrate the initial position estimation with imperfect distance measurements ( $\sigma_{d}^{2}=0.1$). Observe that, as shown in Figures 13 (c)–(f), the error propagation during the estimation process degrades the estimation accuracy for certain nodes and the proposed refinement schemes ( $\sigma_{ɛ}^{2}=0.5$) have limited capability to improve the performance of position estimation. Therefore, a fundamental problem when locating sensors in a network is to estimate the distance between pairs of sensors since accurate location estimates highly rely on precise distance measurements.

### 5.5. An Example of Merging Two Clusters

**Figure 14.**
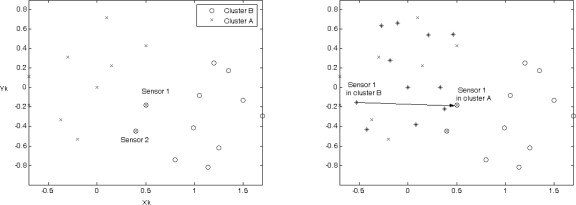
The global coordinate system of cluster *A* and cluster *B* (left); the local coordinate systems of cluster A and cluster B and the shifting direction of cluster B (right).

Figure 14 (left) shows the structure of two clusters generated from the initialized sensors *A* and *B*. There are two shared border sensors, sensor 1 and sensor 2, in the overlapping area. Figure 14 (right) shows the position estimations of local coordinate systems. Note that applying the estimation procedures, these two clusters are generated from the initialized sensors *A* and *B*, which are located at the origin in their local coordinate systems.

**Figure 15.**
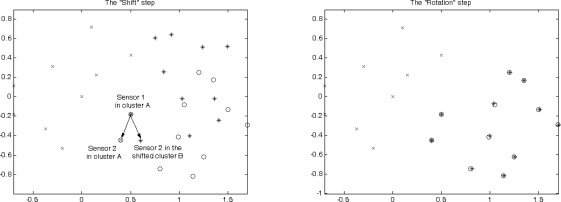
The shifted cluster B and the rotation angle *θ_merge_* (left); the positions of the sensors in the reoriented cluster match the corresponding positions in the coordinate system of sensor A using the rotation matrix *R_merge_* (right).

Once the border sensors have the information of local coordinate systems from two clusters, they transmit *Merge* signals. The effect will be to reorient the cluster centered on sensor *B*. Therefore, sensor 1 uses its own information and the merge signal received from sensor 2 to calculate the adjustment information. Then sensor 1 transmits an *Adjust* message to the sensors in the reoriented cluster to update their positions. Figure 15 (left) depicts the movement of the reoriented cluster using the vector $\vec{d_{S}}$ and Figure 15 (right) shows that the positions of the sensors in the reoriented cluster match the corresponding positions in the coordinate system of sensor *A* using the rotation matrix *R_merge_*.

### 5.6. Cooperative Estimation Fusion

In this set of experiments, two cases of the measurement deviation of the known positions are considered: $\sigma_{P}^{2}=0.01$ and $\sigma_{P}^{2}=1$. The initial settings and group topology are illustrated in Figure 5. Without loss of generality, suppose that the prior information for each cooperative scheme is the bounding box (detailed in Section 3.1.) of the estimated sensor using three neighboring sensors (e.g., the three black nodes shown in Figure 5). For the centralized approach, the estimated sensor receives the distance and position information sent from each cooperative member and includes these information into measurement term (3). For the progressive approach, each cooperative group generates *N*_1_ = 250 samples from the bounding box and *N*_2_ = 250 samples from the Gaussian approximation from previous estimation group, which is described is Table 4. For the distributed scheme, each cooperative group generates *N* = 500 samples from the prior distribution (i.e., the bounding box) for particle filtering. Given the distance and position information of the neighboring known sensors, the best possible position measurement of the estimated sensor is obtained by combining 300 typical runs for each cooperative approach.

**Figure 16.**
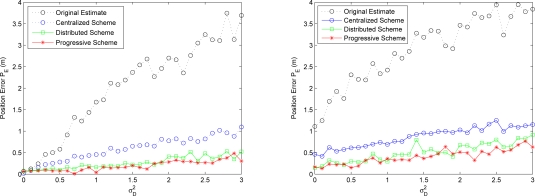
The comparison of position estimation with and without cooperative fusion schemes for different variances of the distance measurement; $\sigma_{P}^{2}=0.01$ (left) and $\sigma_{P}^{2}=1$ (right); the position error $P_{E}^{m}=\left\|P_{\text{true}}^{m}-P_{\text{estimate}}^{m}\right\|$ for sensor *m*.

Observe that in the case of good distance observations and a moderate position deviation, Figure 16 depicts that the estimated sensor may still obtain acceptable position estimate without cooperation. On the other hand, with poor distance observations, the cooperative techniques may be good approaches to improve the estimation accuracy.

The analysis of energy consumption (derived in Section 4.3.) shows that the total energy consumption of these estimation schemes are close when the data of all the sensors are applied. Given an accuracy threshold, the progressive process may terminate and return results without having all the sensors being visited such that computation time and network bandwidth can be reserved. Therefore, the strength of the progressive estimation scheme is the reduction of the amount of communication and the conservation of energy. However, compared with the centralized and distributed schemes, the progressive approach may produce a larger processing delay since it may spend more time on information processing when using the data from all the sensors. Among these three cooperative estimation methods, the distributed scheme may provide an efficient way to weight out the faulty sensors and corrupted observations to suppress the estimation error. This is because if some sensors are faulty or the observations are corrupted, the fusion among all the neighboring sensors (*i.e.*, the centralized scheme) may degrade the estimation performance as shown in Figure 16.

### 5.7. Comparison

This subsection compares the localization algorithms from two perspectives: the algorithm perspective and the network topology perspective. Hence, the probabilistic approximation method (*i.e.*, the particle filter) and the deterministic methods are investigated from the algorithm perspective. Moreover, the proposed hierarchical approach and other cluster-based approaches are discussed from the network topology perspective.

#### 5.7.1. Algorithm Perspective

Several deterministic methods are proposed for sensor localization [7, 9, 60, 61]. For instance, the Min-max method [7], where the main idea is to construct a bounding box for each reference node using its position and distance measurement, and then to determine the intersection of these boxes. Notice that the above methods solve the localization problem with a single estimate; the point algorithm (SPA), where the location estimate is placed at the same position [9]; the centroid algorithm, where the geometric centroid of the positions of the sensors that generates measurements presents the location estimate; the smooth weighted centroid algorithm, where the centroid position computation is weighted by the sensor likelihood models (e.g., the characteristics of the sensors and the measurements) [61]. In contrast, the proposed CHPA method carries along a complete distribution of position estimation.

**Figure 17.**
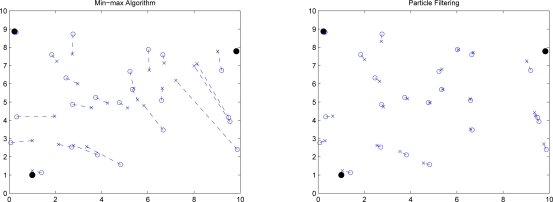
Using min-max and particle filtering for initial position estimation.

The following experiments are conducted in order to depict the weakness and the strength of each method. Figure 17 depicts the initial position estimation using Min-max [7] and particle filtering. Note that “•” represents the reference node, “○” represents the unknown node, and “×” represents the estimated sensor location. Min-max method has the advantage of being computationally cheap, but it requires a good placement for anchors. In Phase I, we use particle filter method with bounding box algorithm to carry out the initial position estimation. Compared with Min-max (Figure 17 (left)), particle filter method Figure 17 (right)) can give more precise location estimation in initial phase. In addition, the distribution of the initial position estimate can be used to do refinement.

**Figure 18.**
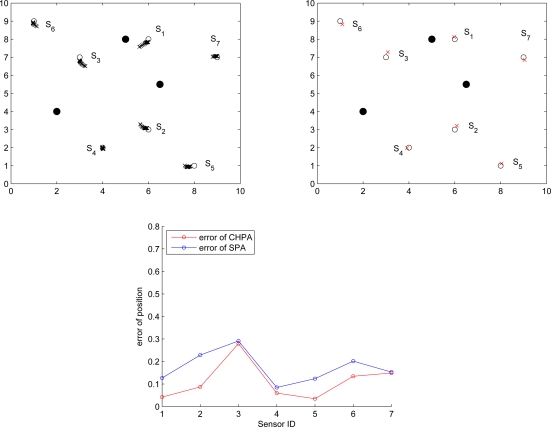
The average position error of each unknown sensor with the measurement noise $\sigma_{d}^{2}=0.01$; the proposed CHPA (top left); the SPA (top right).

**Figure 19.**
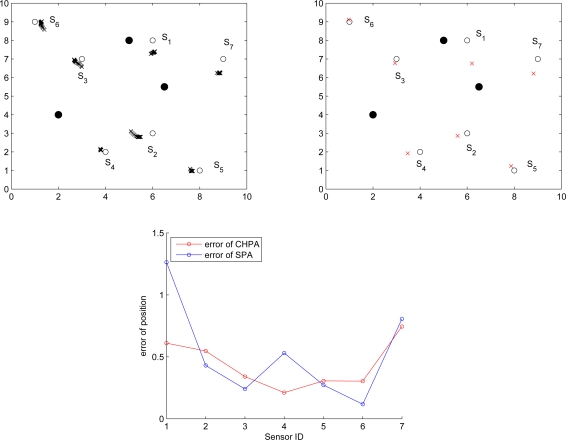
The average position error of each unknown sensor with the measurement noise $\sigma_{d}^{2}=0.1$; the proposed CHPA (top left); the SPA (top right).

Given the errors of distance measurements, Figures 18 and 19 depict the position errors of the proposed CHPA and the SPA [9] schemes. In the absence of measurement noise, the distance between the unknown sensor and the reference nodes defines a circle corresponding to possible sensor locations. Hence, the intersection of at least three circles gives the exact sensor location. However, due to the noisy measurements, these circles do not intersect at the same point. For the SPA scheme, two noisy measurements are applied to obtain two possible locations. Then the estimate of the unknown sensor location is determined by the third distance measurement.

Observe that in Figure 18 with a small distance measurement noise ( $\sigma_{d}^{2}=0.01$), the average estimated positions using the proposed CHPA and the single estimate using the SPA scheme are close. The average position errors of these two schemes nearly fall within 3% of the side length of the square *l* = 10, which suggests that the deterministic localization method (SPA) may be applied to roughly determine the possible sensor location since the influence of the measurement variation on the localization performance is small in this scenario. On the other hand, in Figure 19 with a larger distance measurement noise ( $\sigma_{d}^{2}=0.1$), although the average position errors of these two schemes nearly fall within 10% of the side length of the square, the SPA scheme may not explicitly describe the estimation behavior using a single estimate. In contrast, the proposed CHPA scheme may provide the statistical information of the estimation behavior with a distribution of the estimated location.

As expected, the proposed CHPA algorithm with particle filtering do require more computation time and memory than simpler deterministic position estimation algorithms. However, as shown in [11], the particle filter method is proved to be feasible for location estimation on real devices used in ubiquitous computing. Therefore, the proposed sensor positioning system may be practical to share data from different sensor types and to provide distributional estimates to higher-level services and applications.

#### 5.7.2. Network Topology Perspective

The cluster-based localization approaches are proposed because of the need for scalability and efficiency. Compared with the registration processes in [9] and [44], the computational complexity of the proposed method (detailed in Section 4.3.) is much lower since the calculations are not easy to implement for wireless sensor networks. [18] applies a complex multidimensional scaling (MDS) algorithm [62] to estimate the position of cluster heads. Thus, the cluster members can use clusterheads as reference nodes. Although this approach achieve reasonable accuracy, computational and communication overheads are high. In [19], anchor nodes are deployed for deriving the position of adjacent clusterheads such that this set of nodes with known positions may form a basis for other clusterheads to localize themselves. The problem with manual entry of position information may limit the size and scalability of a sensor network. Therefore, the proposed CHPA approach may provide an efficient way to build up a coordinate system for wireless sensor networks.

## 6. Conclusions

We propose a distributed algorithm for the sensor positioning problem in hierarchical wireless sensor networks. By performing the proposed estimation procedures, a single global coordinate system can be established without GPS sensors using only distance information. In order to elevate the estimation accuracy, the Markov chain Monte Carlo (MCMC) steps may be applied to reduce the estimation error such that the propagation error can be suppressed. In the case of poor observations, cooperative estimation fusion schemes are proposed to complement the measurements of the environment and improve the estimation accuracy. Furthermore, the same basic approach can also solve the tracking problem in which the decentralized sensors combine their information to produce improved estimates of the target location. Therefore, one of the strengths of the approach is that (essentially) the same algorithm can be used to track targets with unknown positions.

There are many other algorithmic questions that would be worthwhile exploring. For example, the resampling method we have used is basic. More advanced techniques may be appropriate, depending on how typical distributions evolve, how many particles should be used, and how does this depend on the number of sensors, their density (in space), etc. Furthermore, we believe there are many ways to improve the performance of the algorithm: (a) by quantifying the trade-offs between amount of communication, speed of computation, and accuracy of the final estimates. (b) by examining alternate ways to “fuse” the received data. For example, once distributional estimates are “shared” between nearby sensors, what are the best ways of incorporating the data? (c) by using a notion of the reliability of the received data. For example, if the “distance” measured between two sensors varies, then this variation suggests an unreliability in the data and hence it should be discounted compared to measurements which are always consistent.

For the proposed measurement solution, trade-offs are found between model complexity, energy consumption, estimation accuracy, and sensible model description in real systems. Future plans will involve generalizing the methods to perform actual measurements to evaluate the performance of the proposed positioning system in ubiquitous computing environments.

## Figures and Tables

**Table 1.** The Particle Filtering Method.

Initialization: Generate a set of random samples *x_k_*(*i*), *i* = 1, 2, . . . , *N* from the prior density at time *k* = 0. Each sample of the state vector is a ‘particle’. Prediction: Each random sample is passed through the state equation to obtain samples from the prior density at time *k* + 1. Thus ${\hat{x}}_{k+1}(i)=\Phi x_{k}(i)+\Gamma w_{k}(i),$ where *w_k_*(*i*) is a sample drawn from the probability density function of the system noise. Measurement Update: The weights of the likelihood function *p*(*z*_*k*+1_|*x̂*_*k*+1_(*i*)) are updated for each sample in the random set *i* = 1, 2, . . . , *N* and the normalized weights are $q_{k+1}(i)=\frac{p(z_{k+1}|{\hat{x}}_{k+1}(i))}{\sum_{j=1}^{n}p(z_{k+1}|{\hat{x}}_{k+1}(j))}$ for each sample. Resampling: Take *N* samples with replacement from the random sample set *x̂*_*k*+1_(*i*), *i* = 1, 2, . . . , *N*, to generate the new sample set *x*_*k*+1_(*i*). Position: The best single estimate of the position is the mean of *x*_*k*+1_(*i*), *x̄*_*k*+1_.

**Table 2.** The Metropolis-Hastings Algorithm.

1. Set *k* = 0 and repeat for *x_k_*(*i*)*, i* = 1, 2, . . . , *N . N* is the number of samples.
2. Draw $x_{k}^{\prime }(i)$ from the proposal density *q*(*x_k_*(*i*), ·).
3. Set *u* to a draw from a *U*(0, 1) distribution.
4. Acceptance probability: $$\alpha (x_{k}(i),x_{k}^{\prime }(i)=\text{min}\left\{1,\frac{\pi (x_{x}^{\prime }(i))q(x_{k}^{\prime }(i),x_{k}(i))}{\pi (x_{k}(i))q(x_{k}(i),x_{k}^{\prime }(i))}\right\},$$
where π(·) is the target density from which samples are desired.
5. **If** (*u* ≤ Acceptance Probability)
accept proposal and set $x_{k+1}(i)=x_{k}^{\prime }(i)$.
**else**
reject proposal and set *x_k_*_+1_(*i*) = *x_k_*(*i*).
**end**
6. Return the values {*x*_*k*+1_(1), *x*_*k*+1_(2), . . . , *x*_*k*+1_(*N*)} and set *k* = *k* + 1.

**Table 3.** The Message Types for Communication.

*Local* - The target sensor, say sensor *m*, broadcasts a *Local* message to build a local coordinate system. *Info* - When a sensor has information to share, it can broadcast a *Info* signal to its neighboring sensors. *Merge* - A *Merge* signal contains position information of the shared border sensor. It is sent when the sensor has location information from two coordinate systems. *Adjust* - *Adjust* messages contain instruction that all sensors in the cluster must update their coordinates to reflect the merging of the clusters.

**Table 4.** The Progressive Estimation Methodology

Initialization: Group *j*+1 generates a set of random samples *x_k_*(*i*), *i* = 1, 2, . . . , *N* from the bounding box of the estimated sensor (*N*_1_ samples) and the estimated Gaussian posterior density function of estimation group *j* (*N*_2_ samples) at time *k* = 0. *N* = *N*_1_ + *N*_2_. Prediction: For *i* = 1, 2, . . . , *N*, sample from *p*(*x*_*k*+1_*|x_k_* = *x_k_* (*i*)) to have ${\left\{{\hat{x}}_{k+1}(i)\right\}}_{i=1}^{N}$. Measurement Update: Obtain the respective normalized weights by $$q_{k+1}(i)=p(z_{k+1}|{\hat{x}}_{k+1}(i))/\sum_{j=1}^{n}p(z_{k+1}|{\hat{x}}_{k+1}(j))$$ Position: Compute the mean *μ*_*k*+1_ and covariance ∑_*k*+1_ as $$\begin{array}{l} \mu_{k+1}=\sum_{i=1}^{N}q_{k+1}(i)x_{k+1}(i)\\ \sum_{k+1}=\sum_{i=1}^{N}q_{k+1}(i)\;(\mu_{k+1}-x_{k+1}(i))\;{\left(\mu_{k+1}-x_{k+1}(i)\right)}^{H} \end{array}$$ and forward these partial information to the next estimation group *j*+2.

**Table 5.** The Distributed Estimation Fusion.

1. The target sensor, say sensor *m*, broadcasts a fusion message to form the neighboring Bayesian measurement groups.
3. Sensor *m* collects the estimation information from the neighboring multi-Bayesian groups.
4. Use the Mahalanobis distance to test the individual utility measure.
**if** (the utility measure 𝒰*_mℓ_*1)
cooperate with the local estimate with $\omega_{m\ell }=\frac{\frac{1}{{𝒰}_{m\ell }}}{\sum_{k\in U_{s}}\frac{1}{{𝒰}_{mk}}}$, where *U_s_* is the index set of the neighboring estimates that pass the utility test.
**else**
ω*_mℓ_* = 0 (*i.e.*, discard that group estimate)
**end**
5. When the local estimate and the group estimates are non-coherent
(1) choose the estimate which has more confidence (less variance) or
(2) exclude the local estimate and fuse the group estimates with the CI method.
